# Supplementary material for: Hybrid Models and Biological Model Reduction with PyDSTool
Source: PLoS Comput Biol. 2012 Aug 9;8(8):e1002628. doi: 10.1371/journal.pcbi.1002628 (PMC3415397; doi:10.1371/journal.pcbi.1002628)
Supplement: Text S4 — Complete source code for the PyDSTool package (version 0.88.120504). Includes API documentation and help files linking to web pages. This file is identical to the current public release on Sourceforge.net. (ZIP) [file pcbi.1002628.s004.zip › PyDSTool/html/PyDSTool.Toolbox.optimizers.line_search.golden_section.GoldenSectionSearch-class.html]

xml version="1.0" encoding="ascii"?


PyDSTool.Toolbox.optimizers.line\_search.golden\_section.GoldenSectionSearch


| Home | Trees | Indices | Help | PyDSTool |
| --- | --- | --- | --- | --- |

Package PyDSTool ::
Package Toolbox ::
Package optimizers ::
Package line\_search ::
Module golden\_section ::
Class GoldenSectionSearch

[hide private]

[frames] | no frames]

# Class GoldenSectionSearch

source code

```
object --+
         |
        GoldenSectionSearch
```

---

Line Search with the golden section method


|  |  |
| --- | --- |
| Instance Methods | [hide private] |

|  |  |
| --- | --- |
| \_\_init\_\_(self, min\_alpha\_step, alpha\_step=1.0, \*\*kwargs)  Needs to have : | source code |

|  |  |
| --- | --- |
| \_\_call\_\_(self, origin, function, state, \*\*kwargs)  Returns a good candidate Parameters : | source code |

**Inherited from `object`**:
`__delattr__`,
`__getattribute__`,
`__hash__`,
`__new__`,
`__reduce__`,
`__reduce_ex__`,
`__repr__`,
`__setattr__`,
`__str__`


|  |  |
| --- | --- |
| Properties | [hide private] |

**Inherited from `object`**:
`__class__`


|  |  |
| --- | --- |
| Method Details | [hide private] |

|  |  |
| --- | --- |
| \_\_init\_\_(self, min\_alpha\_step, alpha\_step=1.0, \*\*kwargs)  *(Constructor)* | source code |

Needs to have :

- a minimum step size (min\_alpha\_step)

Can have :

- a step modifier, a factor to modulate the step (alpha\_step = 1.)

Overrides: object.\_\_init\_\_

|  |  |
| --- | --- |
| \_\_call\_\_(self, origin, function, state, \*\*kwargs)  *(Call operator)* | source code |

Returns a good candidate Parameters :

- origin is the origin of the search
- function is the function to minimize
- state is the state of the optimizer

  


| Home | Trees | Indices | Help | PyDSTool |
| --- | --- | --- | --- | --- |

|  |  |
| --- | --- |
| Generated by Epydoc 3.0.1 on Fri May 4 15:24:09 2012 | http://epydoc.sourceforge.net |
